# Supplementary material for: The Impact of Biomedical Engineering on the Development of Minimally Invasive Cardio-Thoracic Surgery
Source: J Clin Med. 2021 Aug 28;10(17):3877. doi: 10.3390/jcm10173877 (PMC8432110; doi:10.3390/jcm10173877)
Supplement: Supplementary file 1 [file jcm-10-03877-s001.zip › jcm-1318950-supplementary.pdf]

Supplementary Materials

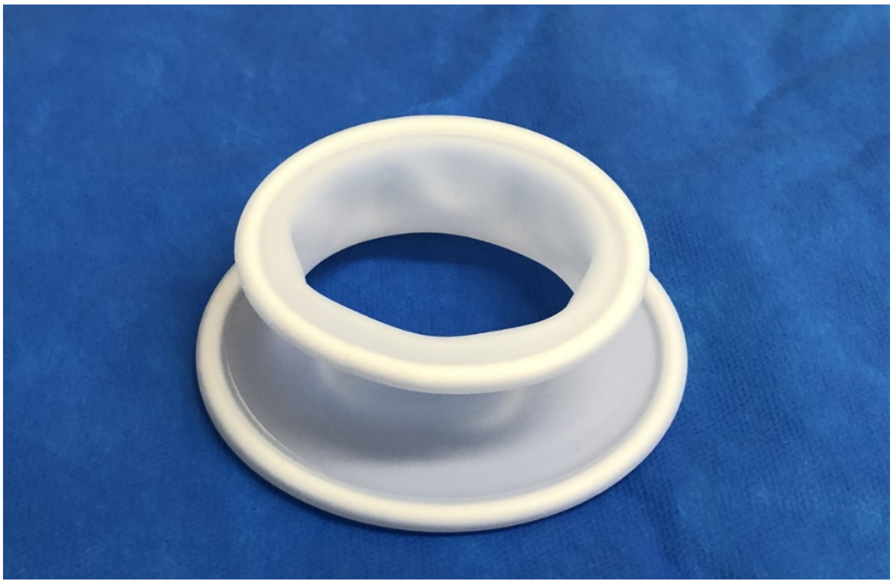

Figure S1. Soft tissue retractor: self-expanding silicone tissue retractor, (Geister Medizinktechnik, Tuttingen, Germany) improves visualization through operational ports.

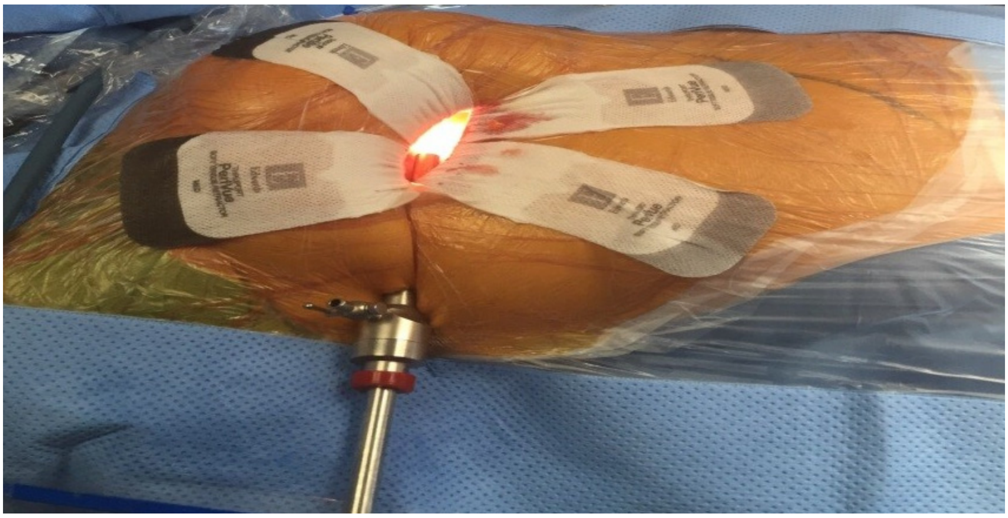

Figure S2. Working port, additional camera, and insufflation port are the only access points needed during minimally invasive mitral surgery. Prosthetic material and instruments are inserted through the 4–5 cm skin incision. This self-expanding or a manually expanded soft tissue improves visualization with minimum tissue trauma.

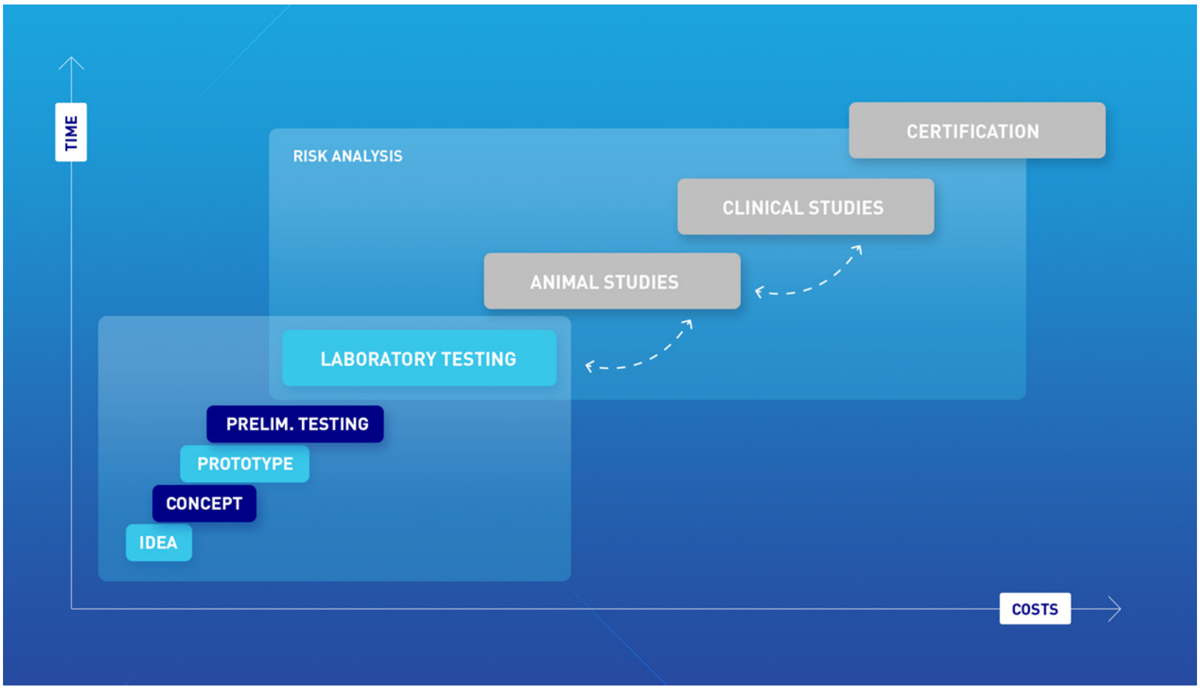

Figure S3. This graph presents the essential device development stages from idea to certification and subsequent commercial application. Each stage is represented in terms of the intensity and costs of research. Concept and laboratory testing with a focus on and collaboration with users was faster compared to studies with animals and patients. Nevertheless, too often the belief in an exceptional technical solution results in a fruitless jump to animal and first-in-man experiments without sufficient critical biomedical engineering testing. While hoping to pass the certification thresholds and satisfy investors, the latter has become more difficult under the recent European Medical Device Regulation (MDR).
